# Calcified Atherosclerosis and Cancer of Low Malignancy

**DOI:** 10.1038/bjc.1963.76

**Published:** 1963-12

**Authors:** A. Elkeles

## Abstract

**Images:**


### CALCIFIED ATHEROSCLEROSIS AND CANCER OF LOW MALIGNANCY

A. ELKELES

*From the Diagnostic X-ray Department of the Prince of Wales's General Hospital and Metropolitan Hospital, London*

Received for publication August 26, 1963

IN this study the term " low malignancy " is designated to cancer which shows a slow rate of growth. This applies either to the primary neoplasm itself or to tumours which after a long period of quiescence lead to secondary spread of slow progression. It also includes malignant neoplasms which after operation show no recurrence during many years of observation.

The degree of malignancy of tumours has mainly been investigated by their histological classification and by the correlation of their microscopical structure with their clinical behaviour. However, these methods of investigation cannot, as yet, explain why malignant new growths with a similar microscopical structure may vary considerably in their biological activity. Latent carcinoma has been found in the prostate (Hirst and Bergman, 1954), lung, kidney, thyroid and elsewhere (Gatch and Culbertson, 1952). These tumours are indistinguishable histologically from active clinical cancers (Franks, 1956). Thus, the concept is gaining ground that the development of clinical cancer and its biological behaviour do not entirely depend on the presence and the microscopical structure of cancer cells but also on an environment conducive to their survival and reproduction. It is, therefore, of importance to search for any possible factors in tissues or organs which may have a retarding effect on the development and the biological activity of cancer.

In previous investigations (Elkeles, 1956, 1959) it was found that with few exceptions, e.g. cancer of the respiratory tract, calcified atherosclerosis of the abdominal aorta shown by radiography, is significantly less common in patients with carcinoma than in control cases of the same age groups. On this evidence the concept of a negative calcified atherosclerosis-cancer relationship was advanced. It was suggested that a biochemical system with an increased affinity of the lipoids and proteins to calcium leads to calcified atherosclerosis and to relative immunity to cancer. A biochemical system with lack of affinity to calcium is responsible for the absence of calcified atherosclerosis in advancing years and is a potential factor in carcinogenesis.

In the course of these investigations a series of patients was observed with cancer of low malignancy. In view of the negative calcified atherosclerosis-cancer relationship it seemed of interest to investigate the incidence and the severity of calcified lesions in the abdominal aorta in these patients.

### *The Study*

This follow-up study refers to 23 patients between the ages of 53 and 79 years, in whom the diagnosis of cancer was established by operation and histology. Radiographs of the abdominal aorta were taken at follow-up examinations. A

classification of the calcified atheromatous lesions was attempted by designating a thin linear plaque about 3–5 cm. long as +, 10–12 cm. long or parallel linear shadows as ++, and more extensive lesions involving almost the whole length of the abdominal aorta as +++.

The age at the time of the diagnosis, sex, site of cancer, years of observation, the outcome when known and the degree of calcification of the abominal aorta are shown in Table I.

TABLE I

| Case number | Sex and age | Site of cancer | Years of observation | Outcome | Degree of calcification of the abdominal aorta shown by radiography |
|---|---|---|---|---|---|
| 1 | M. 70 | Kidney | 14 | died | +++ |
| 2 | M. 74 | Kidney | 5½ | died | +++ |
| 3 | M. 66 | Prostate | 6 | — | +++ |
| 4 | F. 63 | Breast | 11 | died | +++ |
| 5 | F. 59 | Stomach | 7 | alive | +++ |
| 6 | F. 75 | Colon | 8 | died | +++ |
| 7 | F. 71 | Breast + bladder | 10 | alive | +++ |
| 8 | F. 61 | Breast | 15 | alive | +++ |
| 9 | F. 75 | Breast | 8 | died | +++ |
| 10 | F. 56 | Stomach | 6 | died | ++ |
| 11 | F. 69 | Breast | 7 | died | +++ |
| 12 | F. 60 | Breast | 13 | died | +++ |
| 13 | F. 79 | Breast | 7 | died | +++ |
| 14 | M. 72 | Colon | 8 | died | ++ |
| 15 | F. 60 | Colon | 11 | — | ++ |
| 16 | F. 59 | Breast | 11 | alive | + |
| 17 | F. 67 | Colon | 7 | — | ++ |
| 18 | F. 53 | Uterus + breast | 13 | died | ++ |
| 19 | F. 62 | Cervix | 9 | died | +++ |
| 20 | M. 68 | Stomach | 7 | alive | +++ |
| 21 | F. 61 | Uterus | 11 | alive | ++ |
| 22 | F. 67 | Breast | 10 | died | ++ |
| 23 | F. 64 | Breast | 10 | died | ++ |

Cases 1–7 are briefly reported because they illustrate the main features observed in this series of cancer patients. In addition an 8th case, a female aged 32, with cancer of the breast, not included in Table I, is reported for its special interest with regard to the problem of low malignancy.

### *Illustrative Case-histories*

*Case* 1

A male, aged 70, had his right kidney removed for hypernephroma in 1944. He complained of pain in the left chest in 1953. A chest radiograph showed secondaries in the lungs and in the left 7th rib (Fig. 1). He received palliative X-ray treatment to the 7th rib. Contrary to expectation the patient improved and remained comfortable with the exception of occasional attacks of cough with fever. He attended the out-patient department regularly, and serial radiographs of the chest were taken. Some of the secondaries were seen to regress, others progressed slowly (Fig. 2 and 3). The patient died at home in 1958 at the age of 84, 14 years after surgical removal of the hypernephroma and 5 years after the extensive secondaries in the lungs had been found. A radiograph of the abdominal aorta taken in 1953 showed extensive calcification, and re-examination 5 years later, shortly before the patient's death, revealed no diminution of the calcified lesions.

In this patient the secondaries in the lungs were not only of low biological activity but also showed evidence of spontaneous partial regression.

*Case* 2

A male, aged 74, noticed blood in his urine in 1953. An intravenous pyelogram was suggestive of carcinoma of the right kidney. At operation a massive carcinoma of the right kidney was found with involvement of the inferior vena cava. The tumour was considered inoperable. No further therapy was given except for occasional blood transfusions to remedy the anaemia caused by persistent haematuria. The patient died in 1958 of cerebral haemorrhage. Postmortem examination showed complete destruction of the right kidney by malignant new growth. There was no evidence of either further local spread of the tumour or of metastases. The abdominal aorta and the iliac arteries were densely calcified.

*Case* 3

A male, aged 66, complained of dribbling and inability to micturate in 1953. A retropubic prostatectomy was performed. Histology revealed adenomatous hyperplasia of the prostate and areas of poorly differentiated adenocarcinoma. The patient was followed up over a period of 6 years, and no clinical or radiological evidence of secondaries was found.

This is an example of latent cancer of the prostate which in spite of the presence of poorly differentiated cancer cells did not proceed to clinical cancer. This patient showed extensive calcification of the abdominal aorta.

*Case* 4

A female, aged 63, had a radical mastectomy for carcinoma of the left breast in 1950. Histology revealed a scirrhous spheroidal cell carcinoma. In January 1959 she developed pleural effusion, secondaries in the lumbar spine, the left ischium and in the left upper femur. In 1961 a radiograph of the spine showed complete disintegration of the third lumbar vertebra. The patient died in November, 1961, almost 3 years after pleural effusion and widespread secondaries in the skeletal system had been found. This patient showed no regression of the extensive calcified lesions in her abdominal aorta during the protracted downhill course of her neoplastic disease.

---

EXPLANATION OF PLATES.

FIG. 1.—23.4.1953.—Multiple large and small nodular secondaries in both lungs and at inner chest wall at the level of left 7th rib.

FIG. 2.—10.12.1956.—Partial regression of the large secondary in right lower lung. Some of the smaller secondaries are unchanged in size but show increased density. Marked progression of the secondary at left chest wall.

FIG. 3.—21.7.1958.—Slow progression of the large secondary in left lung ; some of the smaller secondaries are no longer visible.

FIG. 4.—Osteolytic secondaries in left ischium and near lateral margin of left iliac bone.

FIG. 5.—Large amount of granular calcium deposits in costal cartilages.

FIG. 6.—The osteolytic secondaries are calcified and hardly visible.

1

3

2

4

6

5

*Case* 5

A female, aged 59, complained of epigastric pain after meals and of loss of weight in 1956. Barium meal examination revealed a small carcinoma in the cardiac region of the stomach. Total gastrectomy and splenectomy were performed. Histology revealed a keratinizing carcinoma with ulceration. The carcinoma had invaded the muscular coat. The patient is alive and well in 1963. She shows marked calcification of the abdominal aorta and of the entire splenic artery.

*Case* 6

A female, aged 75, had been passing blood, dark in colour, with her motions in 1953. A barium enema showed a carcinoma of the proximal portion of the pelvic colon. At operation an ulcerating, constricting tumour 4 cm. long was found, and hemicolectomy was performed. Histology revealed a moderately differentiated adenocarcinoma infiltrating all coats of the colon. The lymph glands adjacent to the tumour were not invaded. The patient kept well until 1961, when she returned to hospital with obstructive jaundice. Secondary growth in the biliary tract was suspected. The operation revealed dense fibrosis of the common bile duct as the cause of the obstructive jaundice. There was no evidence of any secondaries. The patient died from complications caused by the operation. She showed dense calcification of the entire abdominal aorta, of the splenic and hepatic arteries.

*Case* 7

A female, aged 71, had a left mastectomy for carcinoma of the breast in 1953. Follow-up examinations revealed no clinical or radiological evidence of any recurrence. In 1960, the patient complained of blood in her urine. Cystoscopy showed a large carcinoma of the bladder blocking the left ureter. Biopsy revealed a papillary carcinoma of the bladder with no evidence of invasion of the bladder wall. The lesion was treated by endoscopic cysto-diathermy. The patient is alive and shows no recurrence of her neoplasm. It is of interest to note that both neoplasms in this patient are of low malignancy. Her abdominal aorta is densely calcified.

*Case* 8

A female, aged 32, had a radical mastectomy for undifferentiated carcinoma of the left breast in 1953. She complained of pain in the left hip in 1955. Radiographs showed secondaries in the left pelvis, particularly in the ischial bone (Fig. 4). An antero-posterior radiograph of the dorsi-lumbar spine revealed an unusual amount of calcium deposits in the costal cartilages (Fig. 5). The bony secondaries responded to palliative X-ray therapy and injections of testosterone. On radiographs taken in 1957 the secondaries were found to be calcified (Fig. 6). The patient is well and shows no clinical or radiological evidence of secondaries. It is justified to look for any features in this patient which could have contributed to the exceptionally favourable course of her neoplastic disease. The only striking feature is the unusual amount of calcium deposits in the costal cartilages of this patient.

In studies (unpublished) on the age and sex distribution of calcification of the costal cartilages, I found it to be more common and more pronounced in women. Moreover, it occurs at an earlier age in women than in men. However, the degree of calcification of the costal cartilages shown in this patient at the early age of 34

is uncommon. It is of interest that recently similar changes could be found in another patient, aged 39, who showed prolonged survival from carcinoma of the breast. This observation deserves attention in connection with the concept that an increased affinity of the tissues to calcium may play a part in low malignancy.

In this series of 23 cancer patients, who on an average survived for 9 years after the diagnosis was established, all but one showed extensive calcification of the abdominal aorta.

It is widely assumed that cancer is more malignant in the young than in the old. In a follow-up study of 24 patients between the ages of 65 and 87—14 men and 10 women—with cancer of the stomach, one died in 18 months, the others within a year after the diagnosis was established. None of these patients showed any calcification of the abdominal aorta in radiographs.

This observation indicates that old age by itself without an associated increased affinity of the tissues to calcium does not diminish the biological activity of malignant tumours.

### DISCUSSION

It was suggested that wasting or an altered metabolism, caused by a malignant new growth may lead to decalcification of a previously well calcified aorta (Lancet, 1962 ; Fotopoulos, Crampton and Burkhead, 1962). In his necropsy studies, Wilens (1947) found that lipoid deposits may be withdrawn from arterial deposits when death was preceded by terminal wasting. By contrast, he found that terminal weight loss had little effect on the extent of intimal involvement of the coronary arteries by hyalinized or calcified plaques. Analysis of the aortic series revealed the same results. The latter finding is in agreement with my radiological studies of the abdominal aorta in the living. In chronic gastric ulcer of the aged, which is frequently associated with severe pain leading to poor nutrition and wasting, a very high incidence of extensive calcified lesions in the abdominal aorta was found (Elkeles, 1953). When terminal wasting does not cause decalcification of aortas, is there any evidence that an altered metabolism produced by a malignant new growth can do so? In the course of this investigation patients with active cancerous growth were followed up for a number of years until the terminal stage of their disease. They showed no regression of the calcified lesions in their aortas.

Experimental evidence is accumulating for the significance of calcium in maintaining the stability of chromosomes and of cell membranes. Steffensen (1957) found that the microspores and pollen of plants lacking calcium gave, at least, a 17-fold greater rate of chromosome aberrations than those of plants grown on optimal calcium. From his extensive work on spontaneous and on X-ray induced chromosomal aberrations he arrived at the general hypothesis that chromosomes are inherently more sensitive to breakage at reduced levels of calcium. Jayson (1961) and Jayson and Pickering (1962) made similar observations. Applying these experimental findings to human pathology, it was suggested that they may explain the high incidence of leukaemia in patients with ankylosing spondylitis, who had received X-ray treatment (Elkeles, 1962). It is feasible to assume that X-ray radiation of the calcium depleted vertebral bodies in ankylosing spondylitis leads to increased radiosensitivity and to an increased frequency of chromosome aberrations in the bone marrow, thus predisposing to the development of leukaemia.

Calcium is absent or only present in small amounts in cancer tissue (deLong, Coman and Zeidman, 1950). The uncontrolled behaviour of cancer cells has been explained by their decreased cellular adhesiveness caused by lack of calcium on the cell surface (Coman, 1947, 1954). Lansing, Rossenthal and Kamen (1948) found that the rapidly growing malignancy is characterized by its inability to take up calcium[45] from the circulating blood.

In recent years it has been suggested that neoplasia is the consequence of a two-step process. Tyler (1960) advanced the theory that, first, there must be mutational changes that endow a cell with malignant potentialities and second, an environment permissive of neoplastic expression. From their radio-biological studies Failla (1957) and Henshaw (1957, 1962) suggested a unifying concept of natural ageing and carcinogenesis in man. They assume that, at least in part, somatic mutations may account for both processes. They point out that an increase in the number of normal cells mutated into cancer cells will not necessarily lead to a corresponding increase in the incidence of cancer, because the terrain must be propitious before a tumour can develop. According to Failla (1957) this concept permits a more optimistic outlook in cancer control, since it may be easier to modify the terrain than the cancer, once it has been formed.

The clinical observation of a negative calcified atherosclerosis-cancer relationship in conjunction with the results of the present investigation suggest that, when ageing is associated with an increased affinity of tissues to calcium, it produces a terrain which has a restraining effect on the development and the biological behaviour of cancer.

### SUMMARY

An attempt has been made to approach the problem of low malignancy of cancer by searching for features in the host which may influence the biological behaviour of his tumour.

In a series of 23 patients, aged over 50, with cancer of low malignancy 22 showed extensive calcification in the abdominal aorta in radiographs.

The case histories of 8 patients with cancer of low malignancy are briefly reported.

It is suggested that old age by itself, without an associated increased affinity of the tissues to calcium, does not diminish the biological activity of malignant tumours.

Evidence has been produced against the assumption that terminal wasting or a changed metabolism caused by a malignant new growth may lead to the disappearance of calcium in previously calcified aortas.

Based on the observation of a negative calcified atherosclerosis-cancer relationship and on the results of the present investigation, the concept is advanced that ageing, when accompanied by an increased affinity of the tissues to calcium, produces an environment which has a restraining effect on the development and on the behaviour of cancer.

My thanks are due to my clinical colleagues, especially to Mr. Anthony Green, to the radiographic and clerical staff of the Prince of Wales's General Hospital and the Metropolitan Hospital for their co-operation in carrying out this study. I am indebted to Dr. A. Letchner for the medical history of Case 2.

### REFERENCES

Coman, D. R.—(1947) *Science*, **105**, 347.—(1954) *Cancer Res.*, **14**, 519.
deLong, R. P., Coman, D. R. and Zeidman, I.—(1950) *Cancer*, **3**, 718.
Elkeles, A.—(1953) *Amer. J. Roentgenol.*, **70**, 5, 797.—(1956) *Brit. J. Cancer*, **10**, 247.—(1959) *Ibid.*, **13**, 403.—(1962) *Nature, Lond.*, **193**,, 4820, 1089.
Failla, G.—(1957) *Radiology*, **69**, 23.
Fotopoulos, J. P., Crampton, A. R. and Burkhead, H. C.—(1962) *Ibid.*, **79**, 637.
Franks, L. M.—(1956) *Lancet*, ii, 1037.
Gatch, W. D. and Culbertson, C. G.—(1952) *Amer. J. Surg.*, **135**, 775.
Henshaw, P. S.—(1957) *Radiology*, **69**; 30.—(1962) *Ibid.*, **78**, 523.
Hirst, A. E. jun. and Bergman, R. T.—(1954) *Cancer*, **7**, 136.
Jayson, G. C.—(1961) *Nature, Lond.*, **190**, 144.
*Idem* and Pickering, D. C.—(1962) *Lancet*, ii, 1329.
*Lancet*, ii.—(1962) 1097.
Lansing, A. I., Rosenthal, T. B. and Kamen, M. D.—(1948) *Arch. Biochem.*, **19**, 177.
Steffensen, D.—(1957) *Genetics*, **42**, 3, 239.
Tyler, A.—(1960) *J. nat. Cancer Inst.*, **25**, 1197.
Wilens, S. L.—(1947) *Amer. J. Path.*, **23**, 5, 793.


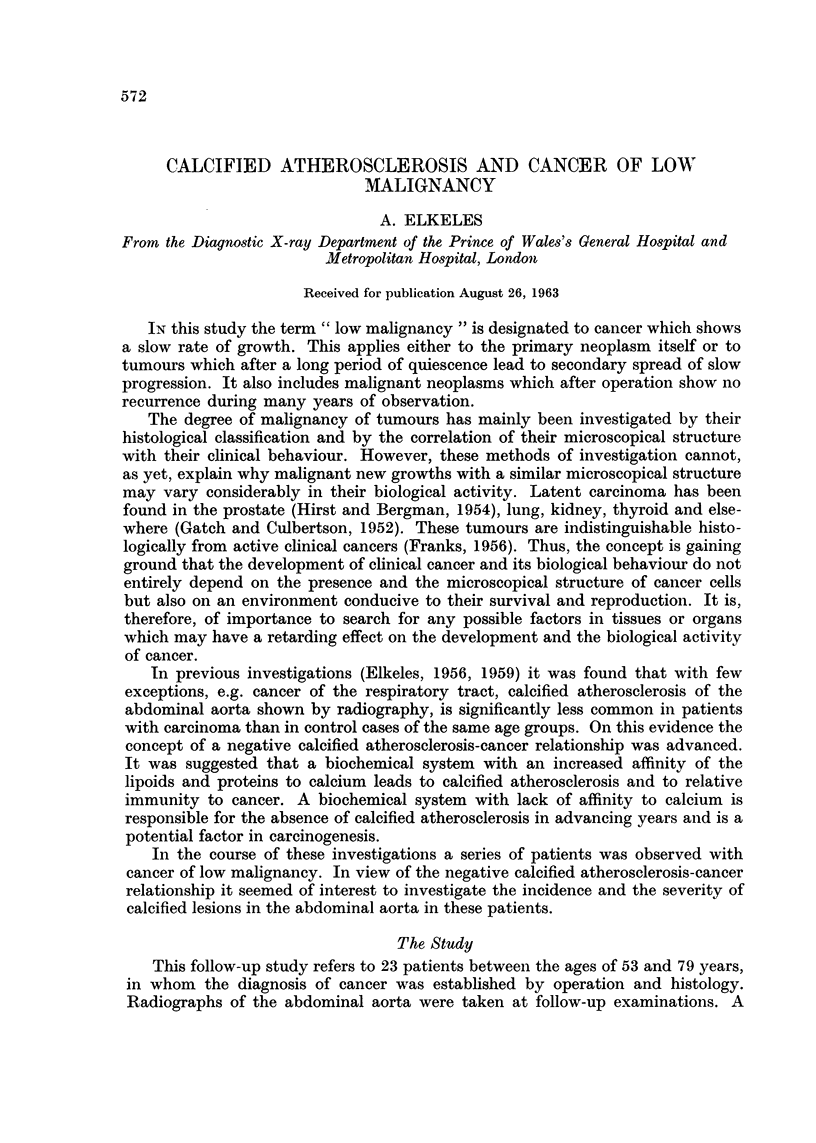

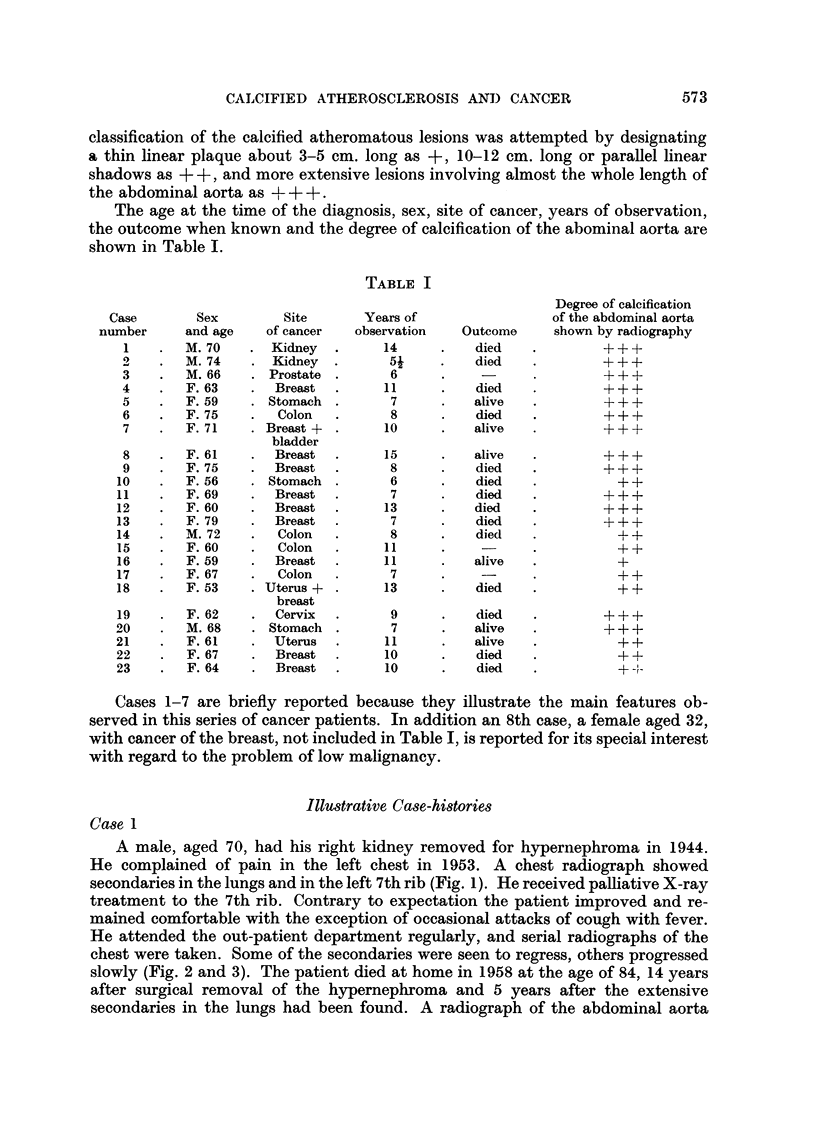

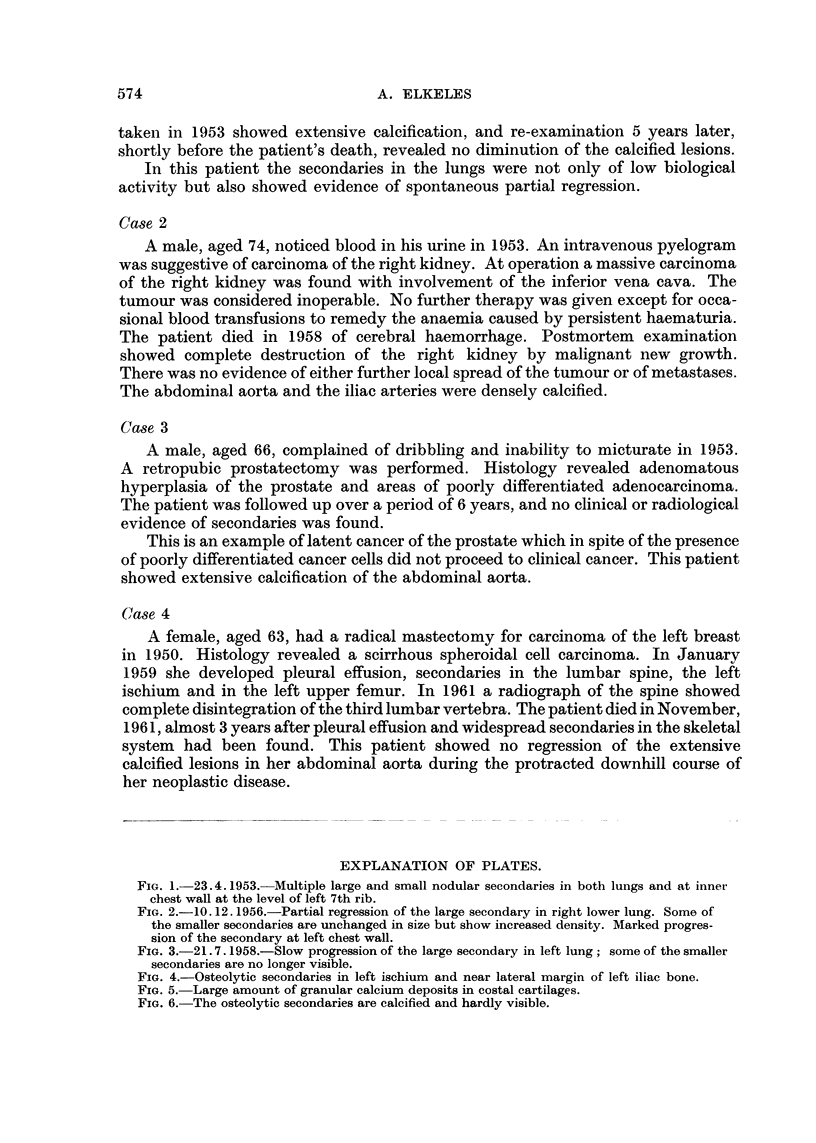

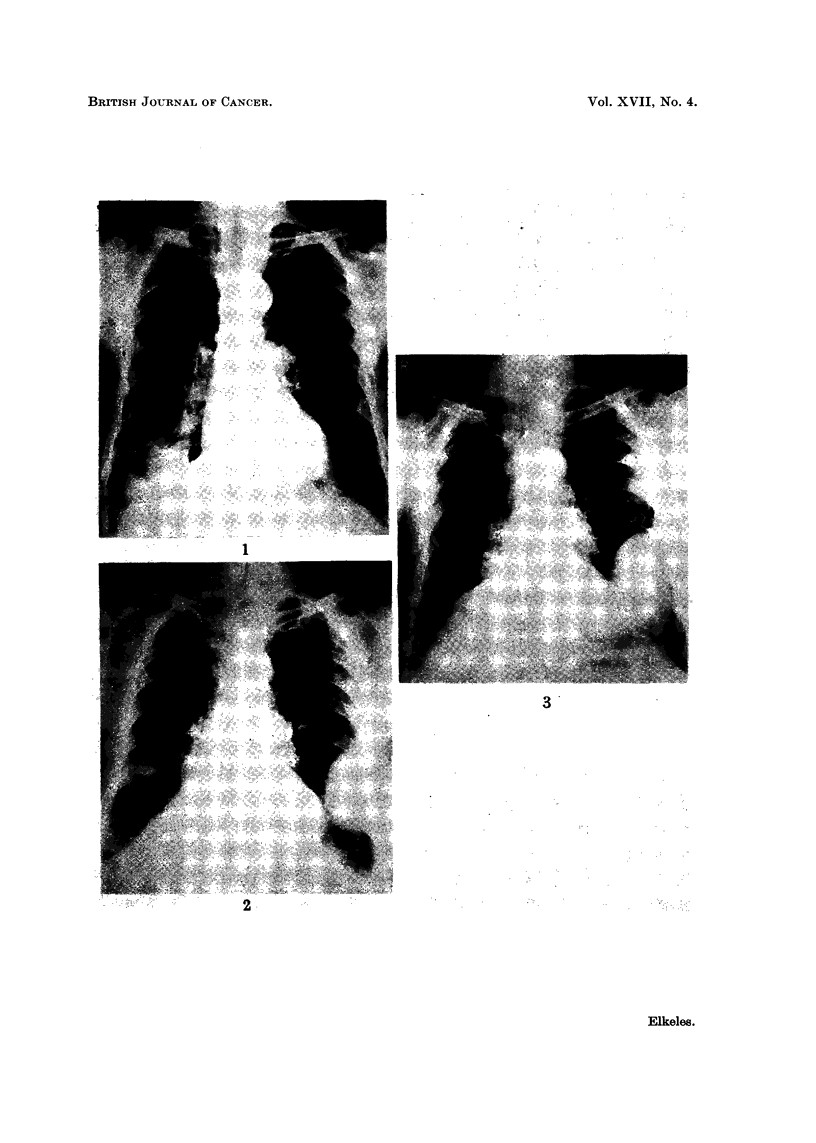

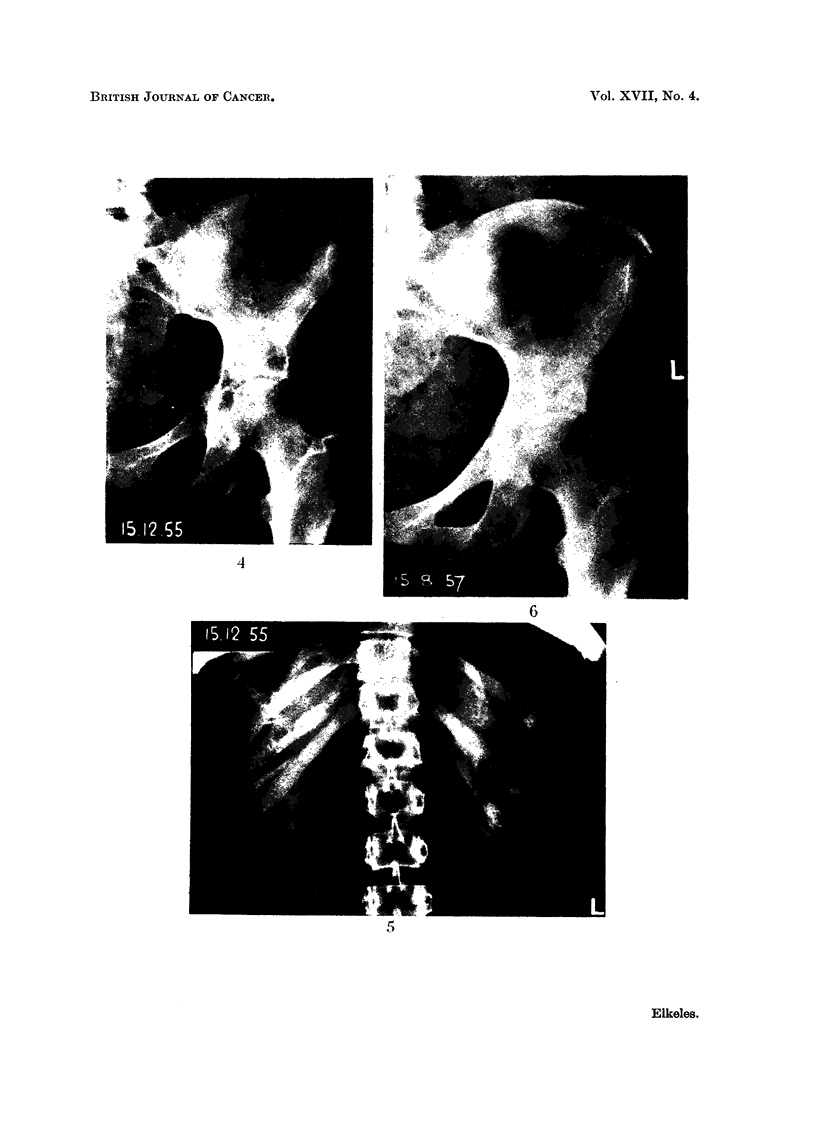

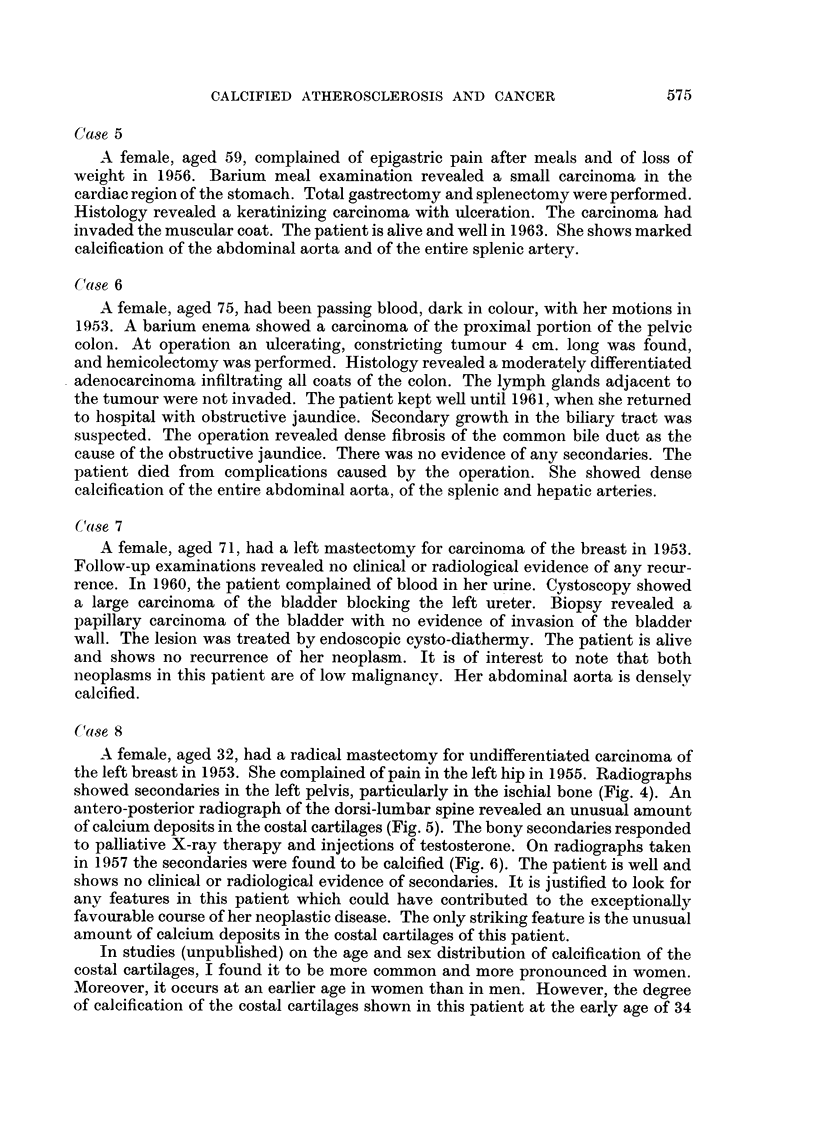

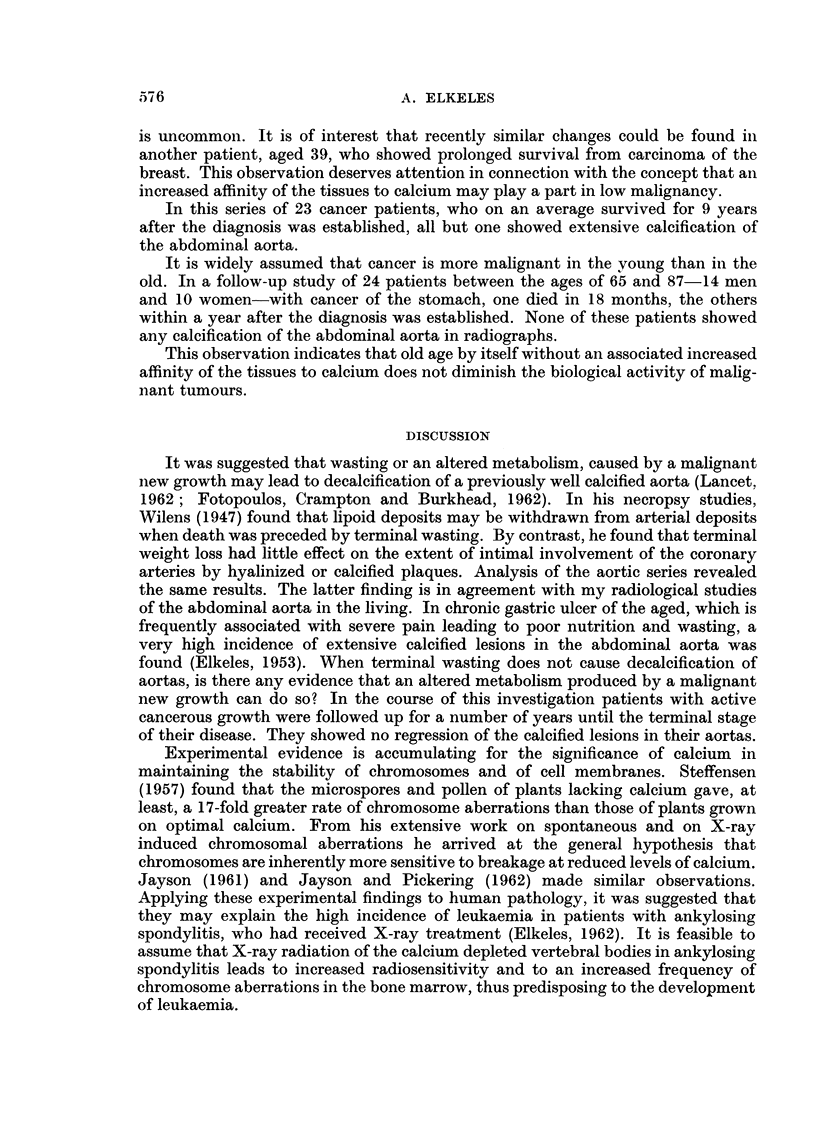

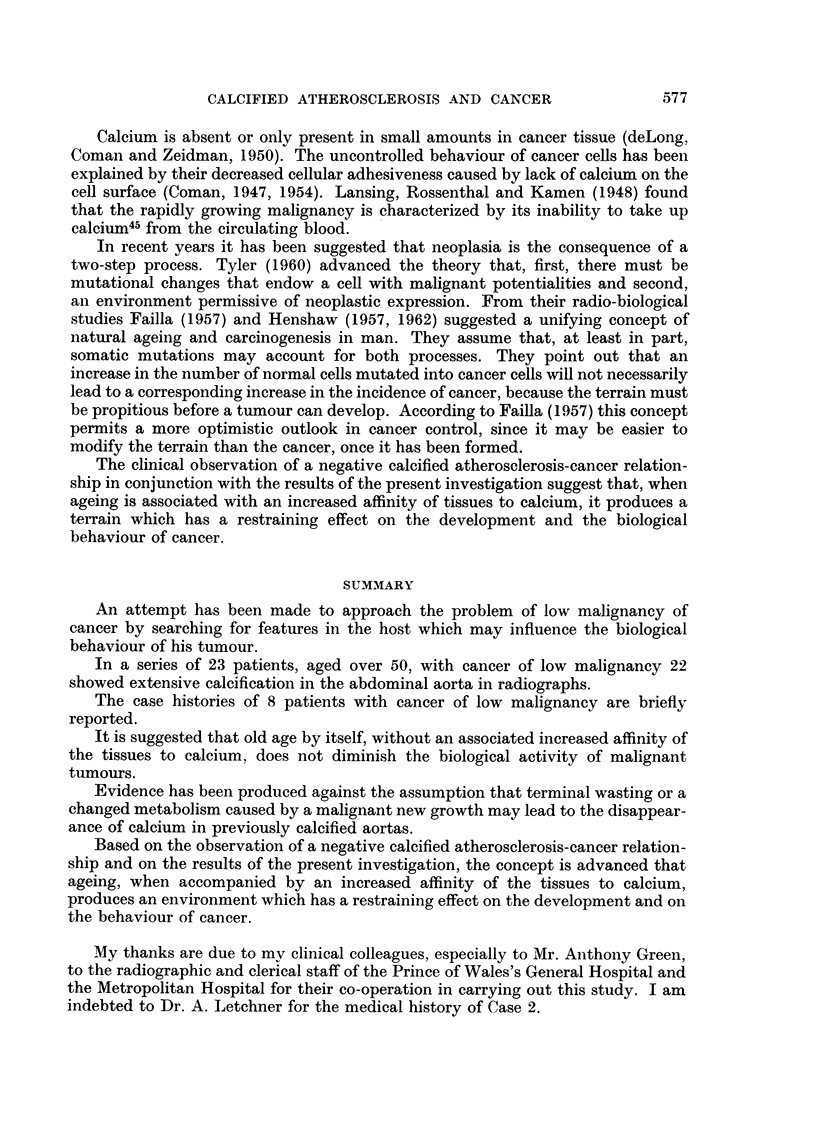

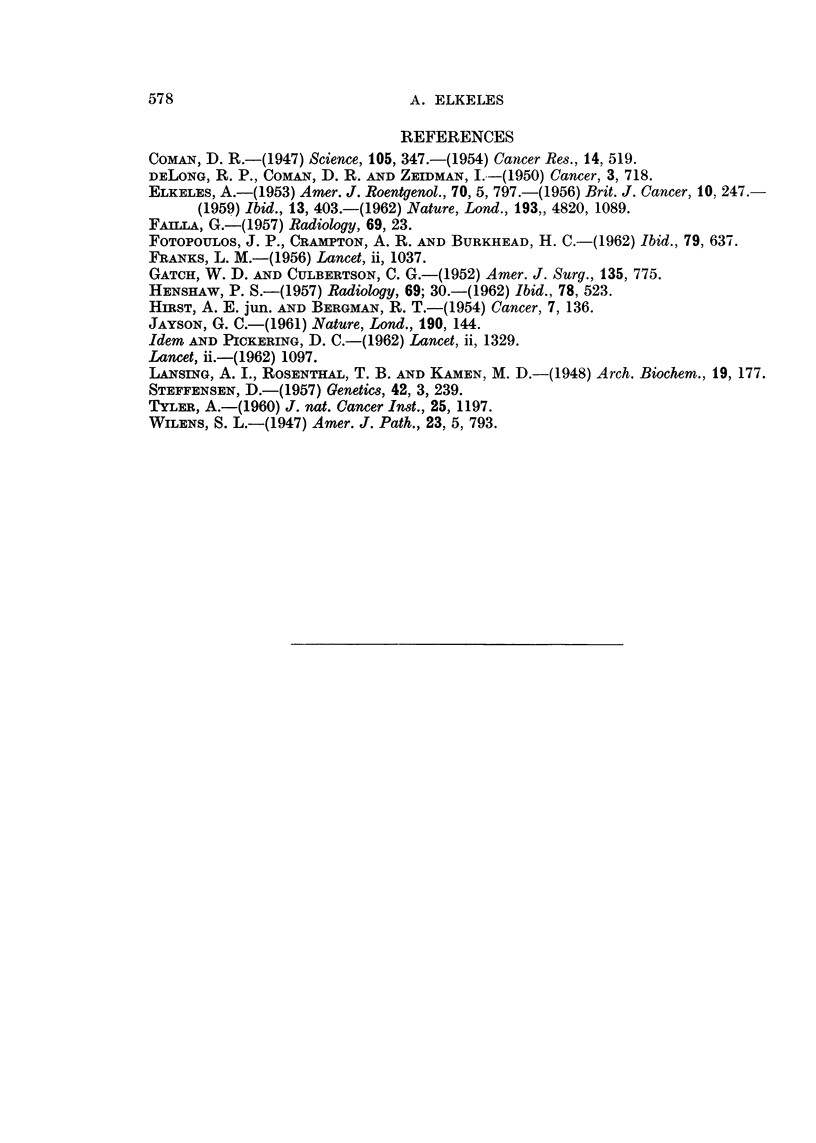


## References

[OCR_00394] ELKELES A. (1962). Calcium and its relationship to radiosensitivity and cancer.. Nature.

[OCR_00399] FOTOPOULOS J. P., CRAMPTON A. R., BURKHEAD H. C. (1962). Calcification of the abdominal aorta as an aid in diagnosis of gastric carcinoma vs. benign ulcer.. Radiology.

[OCR_00402] GATCH W. D., CULBERTSON C. G. (1952). Theories on the treatment of breast cancer and observations on its natural course.. Ann Surg.

[OCR_00403] HENSHAW P. S. (1962). Radiologic life shortening, senescence, and carcinogenesis.. Radiology.

[OCR_00404] HIRST A. E., BERGMAN R. T. (1954). Carcinoma of the prostate in men 80 or more years old.. Cancer.

[OCR_00405] JAYSON G. G. (1961). Bivalent metal ions as the coupling factor between cell metabolism and the rate of cell mutation.. Nature.

[OCR_00411] Steffensen D (1957). Effects of Various Cation Imbalances on the Frequency of X-Ray-Induced Chromosomal Aberrations in Tradescantia.. Genetics.

[OCR_00413] TYLER A. (1960). Clues to the etiology, pathology, and therapy of cancer provided by analogies with transplantation disease.. J Natl Cancer Inst.

[OCR_00415] Wilens S. L. (1947). The Resorption of Arterial Atheromatous Deposits in Wasting Disease.. Am J Pathol.

